# Methodology-Dependent Reversals in Root Decomposition: Divergent Regulation by Forest Gap and Root Order in *Pinus massoniana*

**DOI:** 10.3390/plants14152365

**Published:** 2025-08-01

**Authors:** Haifeng Yin, Jie Zeng, Size Liu, Yu Su, Anwei Yu, Xianwei Li

**Affiliations:** 1 Research Institute of Tropical Forestry, Chinese Academy of Forestry, Guangzhou 510520, China; yhfeng312@163.com (H.Y.); zengjie69@163.com (J.Z.); 2 College of Forestry, Sichuan Agricultural University, Chengdu 611130, China; yuaw006@163.com; 3 Sichuan Academy of Forestry, Chengdu 610081, China; size_leo@126.com; 4 Guangzhou Institute of Forestry and Landscape Architecture, Guangzhou 510405, China; yusu110@163.com

**Keywords:** root decomposition, in situ soil litterbag method, forest gap, fine root order, Masson pine, nutrient cycling, soil–root interaction

## Abstract

Understanding root decomposition dynamics is essential to address declining carbon sequestration and nutrient imbalances in monoculture plantations. This study elucidates how forest gaps regulate *Pinus massoniana* root decomposition through comparative methodological analysis, providing theoretical foundations for near-natural forest management and carbon–nitrogen cycle optimization in plantations. The results showed the following: (1) Root decomposition was significantly accelerated by the in situ soil litterbag method (ISLM) versus the traditional litterbag method (LM) (decomposition rate (*k*) = 0.459 vs. 0.188), reducing the 95% decomposition time (*T_0.95_*) by nearly nine years (6.53 years vs. 15.95 years). ISLM concurrently elevated the root potassium concentration and reconfigured the relationships between root decomposition and soil nutrients. (2) Lower-order roots (orders 1–3) decomposed significantly faster than higher-order roots (orders 4–5) (*k* = 0.455 vs. 0.193). This disparity was amplified under ISLM (lower-/higher-order root *k* ratio = 4.1) but diminished or reversed under LM (lower-/higher-order root *k* ratio = 0.8). (3) Forest gaps regulated decomposition through temporal phase interactions, accelerating decomposition initially (0–360 days) while inhibiting it later (360–720 days), particularly for higher-order roots. Notably, forest gap effects fundamentally reversed between methodologies (slight promotion under LM vs. significant inhibition under ISLM). Our study reveals that conventional LM may obscure genuine ecological interactions during root decomposition, confirms lower-order roots as rapid nutrient-cycling pathways, provides crucial methodological corrections for plantation nutrient models, and advances theoretical foundations for precision management of *P. massoniana* plantations.

## 1. Introduction

Root decomposition is a fundamental process regulating belowground carbon (C) cycling and nutrient return in forest ecosystems [1,2]. This process contributes 4–5 times more C input to the soil than aboveground litterfall [3] and releases 18–45% more nitrogen (N) than aboveground litterfall [4], critically influencing soil organic matter accumulation and ecosystem productivity.

Current methodologies for studying tree root decomposition include static physical approaches (e.g., excavation, soil coring, trenching), dynamic in situ monitoring techniques (e.g., profile wall, minirhizotron), and controlled decomposition experiments (e.g., litterbag method) [5,6]. Among these, the litterbag method has become widely adopted for quantifying root decomposition and nutrient release dynamics due to its standardized protocols and capacity to simulate decomposition environments across different root diameter classes [7]. This method encompasses two primary variants: the traditional litterbag method (LM) and the in situ soil litterbag method (ISLM) [8]. Critically, potential differences may exist between LM and ISLM in their accuracy and applicability for characterizing decomposition dynamics [9,10]. ISLM required thorough mixing of roots with native soil prior to burial, simulating natural root–soil interactions. In contrast, LM maintained physical isolation between roots and surrounding soil, limiting initial microbial colonization (particularly for less motile taxa), and resulting in greater moisture instability [11,12]. However, systematic comparative studies evaluating these two methods within the context of forest management practices remain scarce, leading to uncertainties in methodological selection and data interpretation.

Recent research on forest litter decomposition has progressively shifted focus from aboveground processes to belowground dynamics, yielding significant advances in quantifying driving mechanisms and ecological functions. Particular emphasis has been placed on how plantation management practices regulate belowground carbon–nitrogen (C-N) cycling [13,14,15]. Forest gaps indirectly modulate root decomposition by altering the microenvironment (e.g., increasing soil temperature and moisture, intensifying pH fluctuations), root inputs (e.g., increasing dead root biomass, modifying traits of newly produced roots), and microbial communities (e.g., enhancing bacterial diversity, promoting functional shifts in fungal assemblages) [16,17]. Previous studies indicate dual effects of forest gaps on root decomposition: accelerating processes (e.g., elevating phenolic compound loss rates to 8.5–10.8 mg d−1, reducing litter C/N ratio by 32%) [18,19], while simultaneously exhibiting inhibitory influences (e.g., decomposition rates potentially exhibiting a negative correlation with gap size, oxidative enzyme activities decreasing by 30–50%) [20,21]. Forest gaps also significantly impact C-N cycling (e.g., promoting higher-order root decomposition, increasing ammonium nitrogen content by 20%) [22].

Root order directly governs decomposition rates. Lower-order roots (orders 1–3) decompose over twofold faster than higher-order roots (orders ≥ 4) due to intact cortices, enriched nutrients (e.g., elevated N or phosphorus (P) concentrations), and low lignin content [23,24]. Conversely, higher-order roots exhibit delayed decomposition due to recalcitrant compound accumulation (e.g., lignin/cellulose) and elevated C:N ratios [25]. Environmental and microbial factors further modulate this hierarchical effect. Lower-order roots primarily concentrate in surface soils (0–20 cm) with optimal temperature and moisture, where their decomposition is accelerated by copiotrophic bacteria and mycorrhizal fungi [26,27]. In contrast, higher-order roots predominantly occupy deep soils (>40 cm), characterized by low temperature, hypoxia, and dormance of oligotrophic bacteria, resulting in slower decomposition [28,29].

*Pinus massoniana* Lamb. plantations serve as critical ecological barriers and carbon sinks in southern China [30]. However, large-scale monoculture management has led to declines in soil carbon sequestration capacity, imbalances in nutrient cycling, reduced biodiversity, and soil nutrient depletion, hindering sustainable plantation development [31]. Therefore, investigating root decomposition dynamics in forest gaps is crucial for implementing close-to-nature transformation strategies and regulating C and N cycling in these plantations. This study integrates decomposition methodologies, forest gap treatments, root order, and decomposition time series to analyze root decomposition and nutrient release dynamics in *P. massoniana* roots. We hypothesize the following: (H1) Methodology effects: the in situ soil litterbag method (ISLM) will yield significantly faster root decomposition rates than the traditional litterbag method (LM) due to preserved root–soil–microbe continuity. (H2) Forest gap effects: forest gaps will accelerate root decomposition and nutrient release relative to closed-canopy controls. (H3) Root order interaction: decomposition responses to both methodology (H1) and forest gaps (H2) will differ significantly across root orders, with lower-order roots exhibiting stronger stimulation. The aim of this study is to quantify differential effects between ISLM and LM on root decomposition and nutrient release, and clarify temporal dynamics of root decomposition regulated by forest gap and root order in *P. massoniana*, thereby providing a theoretical framework for close-to-nature plantation management.

## 2. Results

### 2.1. Root Mass Remaining and Decomposition Coefficient

**Figure 1:**
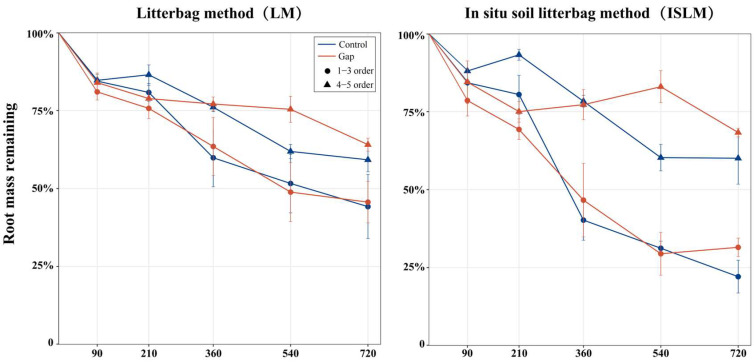
Dynamic changes of root mass remaining during the decomposition process. Control, closed canopy; Gap, 200 m^2^ forest gap.

The root mass remaining of *P. massoniana* differed significantly between both litterbag methods (Figure 1). The mass remaining exhibited greater fluctuation in ISLM than in LM, with larger differences between the Gap and Control treatments in ISLM. From 0 to 360 days, the mass remaining was consistently higher in the Control than in the Gap treatment. However, this pattern reversed from 360 to 720 days, particularly for higher-order roots (orders 4–5). Throughout the experiment, the mass remaining of lower-order roots (order 1–3) was generally lower than that of higher-order roots. By day 720, the root mass remaining was 74.24 ± 9.18% for LM, and 64.08 ± 23.35% for ISLM; 64.56 ± 22.18% for lower-order roots, and 74.77 ± 11.24% for higher-order roots; and 68.93 ± 19.43% for the Control, and 69.40 ± 17.45% for the Gap treatment.

**Figure 2:**
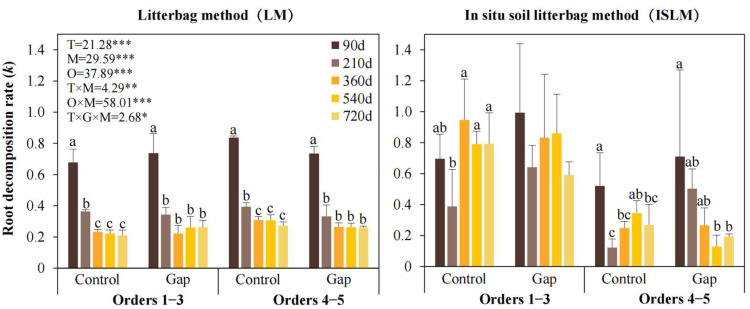
Root decomposition rate (*k*) for different treatments. Control, closed canopy; Gap, 200 m^2^ forest gap. F- and *p*-values of linear mixed-effects models represent the effects of forest gap treatment, decomposition stage, root order, decomposition method, and their interactions on root decomposition coefficient and nutrient release. T, decomposition time; M, decomposition method; G, forest gap treatment; O, root order. *, *p* < 0.05; **, *p* < 0.01; ***, *p* < 0.001. Different lowercase letters above error bars indicate statistically significant values (*p* < 0.05).

The decomposition coefficient (*k*) was significantly higher under ISLM (0.459) than LM (0.188) (Table 1, Figure 2). Consequently, the time required for 95% decomposition (*T_0.95_*) was substantially shorter under ISLM (6.53 years) than LM (15.95 years). The value *k*/year was numerically higher in the Control (*k* = 0.373, *T_0.95_* = 8.04 years) than in the Gap (*k* = 0.274, *T_0.95_* = 10.95 years), although this difference was non-significant. Lower-order roots decomposed significantly faster (*k* = 0.455, *T_0.95_* = 6.59 years) than higher-order roots (*k* = 0.193, *T_0.95_* = 15.54).

When decomposed using ISLM, the value *k*/year was higher in the Control (0.572) than in the Gap (0.346). Conversely, the value *k*/year was higher in the Gap than in the Control under LM. Similarly, under ISLM, the value *k*/year for lower-order roots (0.738) was 3.1 times higher than for higher-order roots (0.180). Under LM, lower-order roots decomposed 16% slower (*k* = 0.171) than higher-order roots (*k* = 0.204) (Table 1).

Decomposition time, decomposition method, and root order significantly influenced *k*, whereas the forest gap treatment exerted no significant effect. Furthermore, significant interaction effects were observed between decomposition time and decomposition method, and root order and decomposition method, as well as among decomposition time, forest gap, and decomposition method (Figure 2).

Under LM, the value *k*/year peaked at 90 days and generally declined to stable over time. Under ISLM, the value *k*/year in general fluctuated considerably, and exhibited a gradual decrease over time only for higher-order roots (4–5) in the Gap treatment. Lower-order roots decomposed faster than higher-order roots under ISLM throughout the experiment (Figure 2).

### 2.2. Different Litterbag Methods

**Figure 3:**
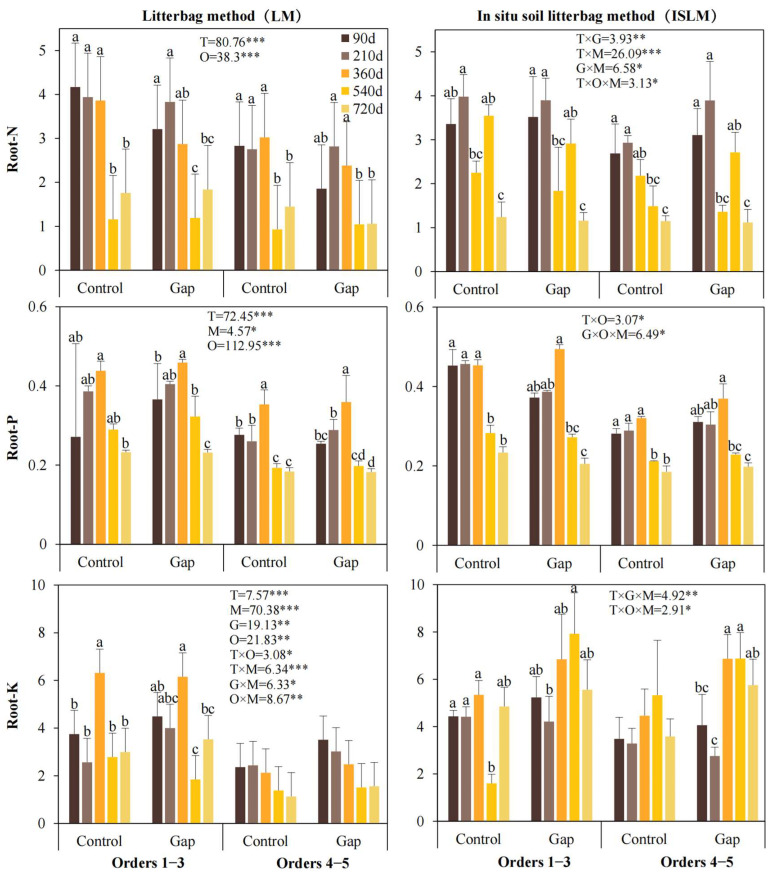
Root nitrogen, phosphorus, and potassium concentrations under different treatments. Control, closed canopy; Gap, 200 m^2^ forest gap. F- and *p*-values of linear mixed-effects models represent the effects of forest gap treatment, decomposition stage, root order, decomposition method, and their interactions on root decomposition coefficient and nutrient release. T, decomposition time; M, decomposition method; G, forest gap treatment; O, root order. *, *p* < 0.05; **, *p* < 0.01; ***, *p* < 0.001. Different lowercase letters above error bars indicate statistically significant values (*p* < 0.05).

Decomposition time and root order significantly influenced root nitrogen (Root-N), phosphorus (Root-P), and potassium (Root-K) concentrations (Figure 3). Overall, concentrations of all three nutrients initially increased and then decreased over time. Decomposition method significantly affected Root-P and Root-K concentrations, with ISLM resulting in significantly higher concentrations of both nutrients than LM. The forest gap treatment only significantly affected Root-K, showing higher concentrations generally in the Gap than in the Control.

Significant interaction effects were detected for Root-N (time × gap, time × method, gap × method, time × order × method), Root-P (time × order, gap × order × method), and Root-K (time × order, time × method, gap × method, order × method, time × gap × method, time × order × method) (Figure 3; all *p* < 0.05).

### 2.3. Link Between Soil Properties and Root Decomposition

**Figure 4:**
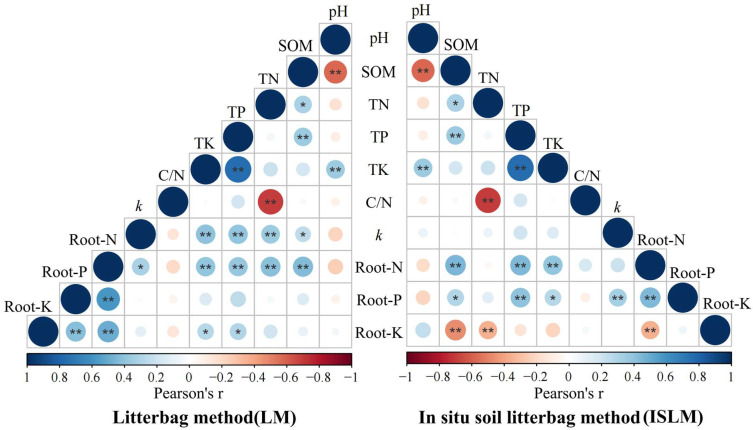
The relationship between soil properties and root decomposition and nutrients in different litterbag methods. *, *p* < 0.05; **, *p* < 0.01. pH, soil pH; SOM, soil organic matter; TN, soil total nitrogen; TP, soil total phosphorus; TK, soil total potassium; *k*, decomposition coefficient; Root-N, root nitrogen; Root-P, root phosphorus; Root-K, root potassium.

Under LM, the value *k*/year and Root-N concentration were significantly positively correlated with contents of soil organic matter (SOM), total nitrogen (TN), total phosphorus (TP), and total potassium (TK); and Root-K concentration was significantly positively correlated with soil TP and TK contents. Under ISLM, the value *k*/year showed no significant correlation with soil properties. Root-N and Root-P concentrations were significantly positively correlated with SOM, TP, and TK contents, while Root-K concentration was significantly negatively correlated with SOM and TN contents. The value *k*/year was significantly positively correlated with Root-N concentration in LM, and with Root-P concentration in ISLM (Figure 4).

## 3. Discussion

### 3.1. Effects of Litterbag Methods on Root Decomposition

The fundamental operational differences between the in situ soil litterbag method (ISLM) and the traditional litterbag method (LM) critically influenced decomposition dynamics. ISLM significantly accelerated decomposition (*k* = 0.459 vs. 0.188 for LM), reducing *T_0.95_* (6.53 vs. 15.95 years) (*p* < 0.05). This enhancement originated from the soil intervention amplifying biologically driven processes [32], as incorporating in situ soil introduced a native microbial community and enzymatic activity, thereby promoting the microbial degradation of lower-order roots [33,34]. Simultaneously, soil fauna disturbance accelerated nutrient release. By simulating microbial and physicochemical contact conditions within the soil matrix, ISLM overcomes the isolation constraints inherent to LM [35].

Nutrient dynamics further underscored methodological differences. The significantly higher root potassium concentration under ISLM (*p* < 0.05) likely reflects enhanced soil ion exchange [36], while the greater fluctuations of root phosphorus release indicate phased microbial nutrient utilization [37], suggesting that ISLM better captures real-world microbial nutrient mining dynamics. Crucially, relationships between decomposition and soil properties were method-dependent. Under LM, the significant positive correlations of decomposition rates (*k*) with contents of SOM, TN, TP, and TK (*p* < 0.05) indicated decomposition governed primarily by soil chemical properties in isolated conditions [38]. Conversely, under ISLM, the lack of a significant correlation between the value *k*/year and soil properties contrasts sharply with the LM results and, instead, the positive correlations of Root-N and Root-P concentrations with SOM and TP contents strongly highlight rhizosphere priming and mycorrhizal mediation as the dominant drivers of nutrient redistribution [39,40]. This shift from abiotic to biotic dominance—driven by microbial network buffering (e.g., enzymatic priming, hyphal bridging)—explains the methodological divergence. These findings align with boreal studies in which in situ conditions accelerated carbon and nitrogen cycling via priming effects [41].

### 3.2. Effects of Forest Gap and Root Order on Root Decomposition

The forest gap treatment exhibited no significant main effect on root decomposition rates, but indirectly regulated the decomposition process through a significant time-dependent interaction (*p* < 0.05). During early decomposition (0–90 days), the higher residual root mass under closed canopy control suggested suppressed microbial activity due to gap-induced microclimate fluctuations in temperature and humidity within forest gaps [42]. Conversely, during the later stage (360–720 days), the significantly lower residual mass in Gap (particularly for orders 4–5) (*p* < 0.05) reflect accelerated lignin degradation by light-enhanced fungal colonization (e.g., ectomycorrhizae) [43], indicating a temporal shift in the primary decomposer community and its drivers.

Notably, forest gap effects were contingent on decomposition method. Under LM, decomposition rates were slightly higher in Gap than Control (*k* = 0.202 vs. 0.174). Conversely, under ISLM, the value *k*/year was significantly higher under Control than under Gap (*k* = 0.572 vs. 0.346) (*p* < 0.05). This reversal demonstrates that soil contact intensity fundamentally modulates environmental factor impacts, likely because ISLM’s enhanced microbial network is more sensitive to microclimate stability under canopy than LM’s isolated system. This methodological interaction underscores the need for careful methodological selection for ecological inference.

Root order exerted a dominant filtering effect on decomposition. Lower-order roots (orders 1–3) decomposed significantly faster (*k* = 0.455) than higher-order roots (orders 4–5; *k* = 0.193), reducing *T_0.95_* by 8.95 years (6.59 years vs. 15.54 years). This disparity arises from the lower C/N ratios and higher soluble compound content in lower-order roots, enhancing microbial bioavailability [41,44]. Previous studies on *P. massoniana* in the Three Gorges Reservoir demonstrated significantly lower residual mass in roots < 0.5 mm in diameter compared to coarser roots (1–2 mm) after 368 days of decomposition [45]. Crucially, ISLM substantially amplified this order-based difference (lower-/higher-order k ratio = 4.1), confirming that in situ conditions intensify decomposition differences driven by root chemistry, likely due to greater microbial access and activity compared to LM.

Root order further modulated nutrient release and interactions. ISLM drastically accelerated lower-order root decomposition. LM modestly suppressed it, underscoring the critical role of soil contact in facilitating decomposition of lower-order roots. Only higher-order roots in Gap exhibited time-dependent value *k*/year decline under ISLM, highlighting their sensitivity to environmental change [1]. Root order significantly influenced the dynamics of all the root nutrients (Root-N, Root-P, Root-K) (*p* < 0.05), specifically regulating K release through its interaction with decomposition method.

### 3.3. Implications for Plantation Management and Research

This study yields three critical insights for *P. massoniana* plantation management and root decomposition science through the interactive effects of decomposition method, forest gap, and root order: (1) Silvicultural practice evaluations should account for temporal scale. Forest gaps facilitated decomposition only during late stages (>360 days), with negligible short-term effects. This is consistent with findings from the Three Gorges Reservoir, where short-term interventions minimally influenced decomposition [46], necessitating long-term perspectives in management. (2) Integrate lower-order roots into nutrient cycling strategies. As rapid-turnover nutrient channels (decomposing 2.36 times faster than higher-order roots), lower-order roots should be integrated into nutrient cycling strategies. Selecting species with rapidly decomposing fine roots in mixed-species plantations could accelerate nutrient cycling, although potential inhibitory effects on specific element release (e.g., N) require attention. (3) Prioritize in situ methods for ecological relevance. The interactions between root order and forest gaps observed under ISLM were masked or reversed under LM. This demonstrates that traditional methods may thus generate misleading ecological inferences, particularly regarding biotic interactions. Future research should therefore prioritize in situ approaches, particularly for studies on soil biotic interactions or environmental gradients.

## 4. Materials and Methods

### 4.1. Study Site

This study was conducted on *Pinus massoniana* plantations at the Dongfanghong Forest Farm, Huaying City, Sichuan Basin, southwestern China (106.77° E, 30.30° N; altitude 600 m). The site experiences subtropical monsoon climate, with a mean annual temperature of 17.5 °C, total annual precipitation of 1128 mm, and average annual humidity of 69%. The soil is classified as Nitisol. Soil pH is 4.2, organic matter content is 41.9 g/kg, total nitrogen content is 1.8 g/kg, total phosphorus is 0.3 g/kg, and total potassium is 15.9 g/kg. The *P. massoniana* plantation was established in the 1980s.

### 4.2. Experimental Design and Sampling

In September 2017, the forest gap experiment employed a randomized block design with three replicates. Within each block, two treatments—200 m^2^ elliptical gap (Gap) and closed canopy (Control)—were randomly assigned, resulting in a total of 6 experimental plots. Each control plot measured 400 m^2^ (20 × 20 m), with all plots spaced > 50 m apart. Forest gaps were created by felling trees at the plot center. The stems, branches, and leaves of all harvested trees were subsequently removed.

*P. massoniana* root samples were collected via excavation from areas adjacent to, but outside, the experimental plots in August 2019. Roots were classified into branch orders 1–5 using the root-order method [47], and categorized into two functional groups: lower-order roots (orders 1–3) and higher-order roots (orders 4–5). Root samples were oven-dried at 65 °C to constant weight, cut into 2 cm segments, and homogenized within each order group. Subsequently, 5 g aliquots of each root group were placed into 15 cm × 15 cm nylon litterbags (mesh size: 0.25 mm). Two decomposition methodologies were implemented. (1) Traditional litterbag method (LM): Litterbag containing roots were buried directly in the primary root distribution zone (5–20 cm depth) without soil addition. (2) In situ soil litterbag method (ISLM): Each litterbag was filled with 100 g of sieved (<2 mm) in situ soil, thoroughly mixed with root samples, and buried in the root distribution zone (5–20 cm depth).

Litterbags were deployed in September 2019 at the center of forest gap plots and within closed canopy control plots. These litterbags were subsequently retrieved at five intervals: December 2019 (90 days), April 2020 (210 days), September 2020 (360 days), March 2021 (540 days), and September 2021 (720 days). This yielded a total of 120 litterbags (6 plots × 2 root groups × 2 decomposition methods × 5 sampling times). Retrieved litterbags were immediately placed on ice and transported to the laboratory. Concurrently with each litterbag retrieval, adjacent soil samples were collected for soil nutrient analysis.

### 4.3. Root Decomposition and Nutrient Analysis

Upon retrieval, roots were carefully separated from adhering soil, cleaned, oven-dried (65 °C) to constant mass, and weighed (±0.0001 g) to determine mass remaining. Dried roots were then ground into a fine powder. The concentrations of root nitrogen (Root-N, g/kg), phosphorus (Root-P, g/kg), and potassium (Root-K, g/kg) were determined [48], specifically using the Kjeldahl method with sulfuric acid digestion and titration for nitrogen, the molybdenum–antimony anti-colorimetric method based on phosphomolybdenum blue complex spectrophotometry at 880 nm for phosphorus, and flame photometry calibrated with KCl standards for potassium.

### 4.4. Soil Properties Analysis

Soil properties were analyzed with following methods [49]: soil pH was measured potentiometrically (Sartorius PP-25 pH meter, Sartorius AG, Göttingen, Germany) in a 1:2.5 soil–water suspension; soil organic matter (SOM, g/kg) concentration was determined by wet oxidation with H_2_SO_4_-K_2_Cr_2_O_7_; soil total nitrogen (TN, g/kg) concentration was estimated by the Kjeldahl method; the C/N was based on ratio of SOC and TN concentration; soil total phosphorus (TP, g/kg) concentration was determined colorimetrically by the molybdenum blue method after digestion with NaOH; and soil total potassium (TK, g/kg) was determined by flame photometry.

### 4.5. Statistical Analysis

All statistical analyses were performed in R (v4.3.1). Data normality (Shapiro–Wilk test) and homogeneity of variance (Levene’s test) were assessed, and non-normal data were log-transformed prior to analyses. Root mass remaining (%) was calculated as *M_t_*/*M*_0_×100%, where *M*_0_ is initial dry mass (g) and *M_t_* is residual dry mass at retrieval time *t* (years). Decomposition coefficient (*k*) was estimated by fitting the Olson exponential decay model, *M_t_*/*M*_0_ = *e*^−*kt*^, where *t* is decomposition time in years (converted from days), and model fitting was performed using nonlinear regression. Half-life (*T_0.5_*) and 95% decomposition time (*T_0.95_*) were calculated as *T_0.5_* = ln (2)/*k* and *T_0.95_* = 3/*k*.

To evaluate effects of forest gap treatment (Gap vs. Control), decomposition stage (5 time points), root order (orders 1–3 vs. orders 4–5), and decomposition method (LM vs. ISLM) on root decomposition coefficient (*k*) and nutrient release (Root-N, Root-P, Root-K), linear mixed-effects models (LMMs) were employed with plot as a random effect. The significance of fixed effects and their interactions were tested using Type III ANOVA. Pearson correlation matrices were constructed for soil properties (SOM, TN, TP, TK) separately for LM and ISLM treatments at each retrieval time, and were visualized using hierarchical clustering heatmaps.

## 5. Conclusions

This study elucidated the core mechanisms through which forest gaps regulate *P. massoniana* root decomposition dynamics by comparing decomposition methodologies. The results demonstrate that the in situ soil litterbag method (ISLM) significantly accelerated root decomposition compared to the traditional litterbag method (LM), reducing the time required for 95% mass loss by nearly 10 years. ISLM fundamentally transformed nutrient release patterns (e.g., elevated potassium concentration) and reconfigured relationships between root decomposition and soil nutrients. Forest gaps regulated decomposition dynamics through temporal phase interactions. They stimulated decomposition during the initial phase (0–360 days) but suppressed it in the later phase (360–720 days), particularly for higher-order roots. Notably, the forest gap effects were fundamentally reversed between methodologies (slight stimulation in under LM vs. significant inhibition under ISLM). This method-dependent reversal cautions against over-reliance on conventional LM approaches, as LM’s physical isolation disrupts microclimatic and microbial mediation of decomposition dynamics. This research demonstrates that conventional LM may obscure critical ecological interactions detectable under ISLM. Our findings provide methodological corrections for plantation nutrient cycling models, highlight the mechanistic validation of lower-order roots as rapid nutrient-cycling pathways, and offer a theoretical foundation for precision management in *P. massoniana* plantations. Specifically, forest gap creation enhances root decomposition to accelerate soil nutrient cycling, validating its efficacy as a key silvicultural intervention; we therefore recommend incorporating forest gap management into plantation transformation strategies.

## Figures and Tables

**Table 1:** Analysis of the decomposition coefficients for decomposition method, forest gap, and root order using the Olson exponential equation regression.

Overall		Regression Equation	R^2^	*k*/year^−1^	*T_0.5_*/year	*T_0.95_*/year
ISLM		y = 0.9488 × *e*^−0.459t^	0.4172	0.459	1.51	6.53
LM		y = 0.8977 × *e*^−0.188t^	0.8202	0.188	3.68	15.95
Control		y = 0.9623 × *e*^−0.373t^	0.4661	0.373	1.86	8.04
Gap		y = 0.8851 × *e*^−0.274t^	0.3317	0.274	2.53	10.95
Orders 1–3		y = 0.9413 × *e*^−0.455t^	0.4477	0.455	1.52	6.59
Orders 4–5		y = 0.9049 × *e*^−0.193t^	0.5752	0.193	3.59	15.54
**Method**	**Treatment**	**Regression Equation**	**R^2^**	***k*/year^−1^**	***T_0.5_*/year**	***T_0.95_*/year**
ISLM	Control	y = 1.0484 × *e*^−0.5723t^	0.5652	0.572	1.21	5.24
	Gap	y = 0.8587 × *e*^−0.3467t^	0.2754	0.346	2.00	8.65
LM	Control	y = 0.8833 × *e*^−0.1746t^	0.8053	0.174	3.97	17.18
	Gap	y = 0.9123 × *e*^−0.2016t^	0.8388	0.201	3.44	14.88
ISLM	Orders 1–3	y = 0.9835 × *e*^−0.7382t^	0.8150	0.738	0.94	4.06
	Orders 4–5	y = 0.9154 × *e*^−0.1807t^	0.4219	0.180	3.84	16.60
LM	Orders 1–3	y = 0.9010 × *e*^−0.1717t^	0.7825	0.171	4.04	17.47
	Orders 4–5	y = 0.8944 × *e*^−0.2045t^	0.9040	0.204	3.39	14.67

LM: litterbag method; ISLM: in situ soil litterbag method. *k*, decomposition coefficient; *T_0.5_*, root half-life; *T_0.95_*, 95% root decomposition time. Control, closed canopy; Gap, 200 m^2^ forest gap.

## Data Availability

All data supporting the findings are included within the article.

## References

[1] Silver W.L., Miya R.K. (2001). Global patterns in root decomposition: Comparisons of climate and litter quality effects. Oecologia.

[2] Li A., Fahey T.J., Pawlowska T.E., Fisk M.C., Burtis J. (2015). Fine root decomposition, nutrient mobilization and fungal communities in a pine forest ecosystem. Soil Biol. Biochem..

[3] Guo L.B., Sims R.E.H. (1999). Litter decomposition and nutrient release via litter decomposition in New Zealand eucalypt short rotation forests. Agric. Ecosyst. Environ..

[4] Gordon W.S., Jackson R.B. (2000). Nutrient concentrations in fine roots. Ecology.

[5] Böhm W. (2012). Methods of Studying Root Systems, Ecological Studies.

[6] Maeght J.L., Rewald B., Pierret A. (2013). How to study deep roots-and why it matters. Front. Plant Sci..

[7] Harmon M.E., Silver W.L., Fasth B., Chen H., Burke I.C., Parton W.J., Hart S.C., Currie W.S., Laundre J., Wright J. (2009). Long-term patterns of mass loss during the decomposition of leaf and fine root litter: An intersite comparison. Glob. Change Biol..

[8] Dornbush M.E., Isenhart T.M., Raich J.W. (2002). Quantifying fine-root decomposition: An alternative to buried litterbags. Ecology.

[9] Sun T., Mao Z., Dong L., Hou L., Song Y., Wang X. (2013). Further evidence for slow decomposition of very fine roots using two methods: Litterbags and intact cores. Plant Soil.

[10] Chen G.-T., Chen Y.-Q., Peng Y., Hu H.-L., Xie J.-L., Chen G., Xiao Y.-L., Liu L., Tang Y., Tu L.-H. (2021). Standard litterbags inestimate early-stage lower-order root decomposition rate in a subtropical forest, China. Plant Soil.

[11] Riutta T., Slade E.M., Bebber D.P., Taylor M.E., Malhi Y., Riordan P., Macdonald D.W., Morecroft M.D. (2012). Experimental evidence for the interacting effects of forest edge, moisture and soil macrofauna on leaf litter decomposition. Soil Biol. Biochem..

[12] Mcclaugherty C., Formation H., Sequestration C. (2014). Plant Litter: Decomposition, Humus Formation, Carbon Sequestration.

[13] Jing Y., Tian P., Wang Q., Li W., Sun Z., Yang H. (2021). Effects of root dominate over aboveground litter on soil microbial biomass in global forest ecosystems. For. Ecosyst..

[14] Saha S., Huang L., Khoso M.A., Wu H., Han D., Ma X., Poudel T.R., Li B., Zhu M., Lan Q. (2023). Fine root decomposition in forest ecosystems: An ecological perspective. Front. Plant Sci..

[15] Carvalho J.I., An J.Y., Tran L.T.N., Carayugan M.B., Kong Y.J., Jo M.S., Hintural W.P., Rahman S.K.A., Lee H.J., Park S.H. (2025). Root decomposition of four temperate species in the Republic of Korea: Associations of root traits and microbial community with root decay. Plant Soil.

[16] Lin N., Bartsch N., Heinrichs S., Vor T. (2015). Long-term effects of canopy opening and liming on leaf litter production, and on leaf litter and fine-root decomposition in a European beech (*Fagus sylvatica* L.) forest. For. Ecol. Manag..

[17] Lyu Q., Luo Y., Dong Y., Xiang Y., Zhao K., Chen G., Chen Y., Fan C., Li X. (2022). Effects of forest gaps on the structure and diversity of soil bacterial communities in weeping cypress forest plantations. Front. Microbiol..

[18] Su Y., He R., Yin H., Guo M., Li X., Fan C., Liu S. (2019). Effects of Cinnamomum septentrionale in hilly area of central sichuan basin on ecological stoichiometry characteristics of Cupressus funebris forest. Acta Ecol. Sin..

[19] Du T., Chen Y., Bi J., Yang Y., Zhang L., You C., Tan B., Xu Z., Wang L., Liu S. (2023). Effects of forest gap on losses of total phenols and condensed tannins of foliar litter in a subalpine forest of western Sichuan, China. Chin. J. Plant Ecology.

[20] Kneeshaw D.D., Bergeron Y. (1998). Canopy gap characteristics and tree replacement in the southeastern boreal forest. Ecology.

[21] Tong R., Ji B., Wang G.G., Lou C., Ma C., Zhu N., Yuan W., Wu T. (2024). Canopy gap impacts on soil organic carbon and nutrient dynamic: A meta-analysis. Ann. For. Sci..

[22] Zhang X., Wang W. (2015). The decomposition of fine and coarse roots: Their global patterns and controlling factors. Sci. Rep..

[23] Guo D., Xia M., Wei X., Chang W., Liu Y., Wang Z. (2008). Anatomical traits associated with absorption and mycorrhizal colonization are linked to root branch order in twenty-three Chinese temperate tree species. New Phytol..

[24] Fan P., Guo D. (2010). Slow decomposition of lower order roots: A key mechanism of root carbon and nutrient retention in the soil. Oecologia.

[25] Guo L., Deng M., Yang S., Liu W., Wang X., Wang J., Liu L. (2021). The coordination between leaf and fine root litter decomposition and the difference in their controlling factors. Glob. Ecol. Biogeogr..

[26] King W.L., Yates C.F., Guo J., Fleishman S.M., Trexler R.V., Centinari M., Bell T.H., Eissenstat D.M. (2021). The hierarchy of root branching order determines bacterial composition, microbial carrying capacity and microbial filtering. Commun. Biol..

[27] King W.L., Yates C.F., Cao L., O’Rourke-Ibach S., Fleishman S.M., Richards S.C., Centinari M., Hafner B.D., Goebel M., Bauerle T. (2023). Functionally discrete fine roots differ in microbial assembly, microbial functional potential, and produced metabolites. Plant Cell Environ..

[28] Siegwart L., Bertrand I., Roupsard O., Duthoit M., Jourdan C. (2022). Root litter decomposition in a sub-Sahelian agroforestry parkland dominated by Faidherbia albida. J. Arid. Environ..

[29] Tang Y., Liu X., Lian J., Cheng X., Wang G.G., Zhang J. (2023). Soil depth can modify the contribution of root system architecture to the root decomposition rate. Forests.

[30] Deng C., Zhang S., Lu Y., Froese R.E., Xu X., Zeng J., Ming A., Liu X., Xie Y., Li Q. (2020). Thinning effects on forest evolution in Masson pine (*Pinus massoniana* Lamb.) conversion from pure plantations into mixed forests. For. Ecol. Manag..

[31] Yang C., Jiang P., Qian Z., Sun J., Wu T., Yang Z., Sun Y., Huang X. (2025). Evolutionary history of mixed tree species improved soil nutrient content of *Pinus massoniana* plantation. Plant Soil.

[32] Ma C., Wang X., Wang J., Zhu X., Qin C., Zeng Y., Zhen W., Fang Y., Shangguan Z. (2023). Interactions of soil nutrients and microbial communities during root decomposition of gramineous and leguminous forages. Land Degrad. Dev..

[33] Liu Y., Zhang A., Li X., Kuang W., Islam W. (2024). Litter decomposition rate response to multiple global change factors: A meta-analysis. Soil Biol. Biochem..

[34] Zhang C., de Pasquale S., Hartman K., Stanley C.E., Berendsen R.L., van der Heijden M.G.A. (2024). The microbial contribution to litter decomposition and plant growth. Environ. Microbiol. Rep..

[35] Lindedam J., Magid J., Poulsen P., Luxhøi J. (2009). Tissue architecture and soil fertility controls on decomposer communities and decomposition of roots. Soil Biol. Biochem..

[36] Ulery A.L., Graham R.C., Goforth B.R., Hubbert K.R. (2017). Fire effects on cation exchange capacity of California forest and woodland soils. Geoderma.

[37] Brown R.W., Chadwick D.R., Bending G.D., Collins C.D., Whelton H.L., Daulton E., Covington J.A., Bull I.D., Jones D.L. (2022). Nutrient (C, N and P) enrichment induces significant changes in the soil metabolite profile and microbial carbon partitioning. Soil Biol. Biochem..

[38] Jones M.E., LaCroix R.E., Zeigler J., Ying S.C., Nico P.S., Keiluweit M. (2020). Enzymes, manganese, or iron? drivers of oxidative organic matter decomposition in soils. Environ. Sci. Technol..

[39] Zhou J., Zang H., Loeppmann S., Gube M., Kuzyakov Y., Pausch J. (2020). Arbuscular mycorrhiza enhances rhizodeposition and reduces the rhizosphere priming effect on the decomposition of soil organic matter. Soil Biol. Biochem..

[40] Li J., Liu Z.F., Jin M.K., Zhang W., Lambers H., Hui D., Liang C., Zhang J., Wu D., Sardans J. (2023). Microbial controls over soil priming effects in chronic nitrogen and phosphorus additions in subtropical forests. ISME J..

[41] Li J., Alaei S., Zhou M., Bengtson P. (2021). Root influence on soil nitrogen availability and microbial community dynamics results in contrasting rhizosphere priming effects in pine and spruce soil. Funct. Ecol..

[42] Arunachalam A., Arunachalam K. (2000). Influence of gap size and soil properties on microbial biomass in a subtropical humid forest of north-east India. Plant Soil.

[43] Wang Z., Tan B., Yang W., Wang Q., Chang C., Wang L., Li H., You C., Cao R., Jiang Y. (2023). Forest gaps accelerate the degradation of cellulose and lignin in decaying logs in a subalpine forest. Eur. J. For. Res..

[44] Iversen C.M., McCormack M.L., Powell A.S., Blackwood C.B., Freschet G.T., Kattge J., Roumet C., Stover D.B., Soudzilovskaia N.A., Valverde-Barrantes O.J. (2017). A global Fine-Root Ecology Database to address below-ground challenges in plant ecology. New Phytol..

[45] Shen Y., Wang N., Cheng R., Xiao W., Yang S., Guo Y., Lei L., Zeng L., Wang X. (2017). Characteristics of fine roots of *Pinus massoniana* in the Three Gorges Reservoir area, China. Forests.

[46] Yang S., Cheng R., Xiao W., Shen Y., Wang L., Guo Y., Sun P. (2019). Heterogeneity in decomposition rates and nutrient release in fine-root architecture of *Pinus massoniana* in the Three Gorges Reservoir Area. Forests.

[47] Pregitzer K.S. (2002). Fine roots of trees: A new perspective. New Phytol..

[48] (1999). Determination of Total Nitrogen, Phosphorus, Potassium, Sodium, Calcium, Magnesium in Forest Plant and Forest Floor.

[49] Gregorich E.G., Carter M.R. (2007). Soil Sampling and Methods of Analysis.

